# A new non-invasive index for the prediction of endotracheal intubation in patients with SARS COVID-19 infection, in the emergency department, pilot study

**DOI:** 10.1186/s12890-023-02435-2

**Published:** 2023-04-21

**Authors:** Germán Devia Jaramillo, Luis Carlos Venegas Sanabria, Carolina Buitrago

**Affiliations:** 1 Emergency Medicine, Hospital Universitario Mayor Méderi, School of Medicine and Health Sciences, Universidad del Rosario, Bogotá, Colombia; 2 Hospital Universitario Mayor Méderi, Bogota, Colombia; 3 School of Medicine and Health Sciences, Universidad del Rosario, Bogota, Colombia

**Keywords:** COVID-19, POCUS, Emergency, Lung ultrasound

## Abstract

**Background:**

In the current context of the SARS COVID-19 pandemic, where the main cause of death is respiratory failure, and since early recognition would allow timely measures to be implemented and probably improve outcomes, it is important to have tools that allow the emergency room to predict quickly and without the use of large resources which will need invasive mechanical ventilation. This study proposes using a new predictive index of noninvasive characteristics, based on the relationship between oxygenation and work of breathing measured by ultrasound-assessed diaphragmatic function, for the need for invasive mechanical ventilation in patients with SARS-COV2 infection who are admitted to the emergency department.

**Methods:**

A prospective predictive cohort study was performed, collecting all patients admitted to the emergency room with respiratory failure (not severe or in imminent respiratory arrest) and a confirmed diagnosis of SARS-CoV-2 pneumonia. Diaphragmatic excursion measurements were taken within the first 24 h after admission to the department. The relationship between diaphragmatic excursion and SAFI was calculated, establishing the ultrasound diaphragmatic excursion So2/FiO2 index (U.D.E.S.I). The index’s performance was determined by analysis of sensitivity, specificity, and area under the curve (AUC).

**Results:**

This pilot study analyzed the first 100 patients enrolled and found in-hospital mortality of 19%, all patients who died required mechanical ventilation, the right index showed a specificity of 82.4% with a sensitivity of 76.9%, likewise for the left index an overall specificity of 90.5% with a sensitivity of 65.3% was found. The ideal cut-off point for the right index is 1.485, and for the left index, the threshold point was 1.856. AUC of the right index is 0.798 (0.676–0.920) and of the left index 0.793 (0.674–0.911), when comparing them no significant differences were found between these values *p* = 0.871.

**Conclusion:**

The relationship of So2/FiO2 and diaphragm excursion measured by both right and left ultrasound could predict the need for mechanical ventilation of the patient with COVID-19 pneumonia in the emergency room and could constitute a valuable tool since it uses noninvasive parameters and is easily applicable at the patient’s bedside. However, a more extensive study is needed to validate these preliminary results.

## Background

The inability of the pulmonary system to meet the metabolic demands of the body is considered a respiratory failure [1]. Clinical assessment of respiratory failure is difficult and often unreliable, except when the patient is in salvage therapy or imminent respiratory arrest [2]. Respiratory failure treatment generally consists of orotracheal intubation and mechanical ventilation. In the current context of the SARS-CoV-2 pandemic, where the main cause of death is a respiratory failure [3], and based on the fact that early recognition of infectious diseases such as sepsis allows timely measures to be put in place and probably improve outcomes [4], It is important to have tools that allow the emergency room to predict, quickly and with little use of resources, the need for invasive mechanical ventilation in patients diagnosed with SARS-CoV-2 infection.

Currently, there are predictors of mechanical ventilation, such as the ROX index, defined as the relationship between pulse oximetry on the fraction of inspired oxygen and respiratory rate, which is validated in patients with acute respiratory failure and pneumonia who were already being treated with high-flow humidified and heated nasal cannula, mainly identifying patients at low risk in whom therapy can be continued after 12 h, however, without a clear definition of the precise time of invasive mechanical ventilation [5]. Other predictors have been used, one of them taken from the criteria published in Berlin in 2012 on the definition of Acute Respiratory Distress Syndrome (ARDS), which is based on the relationship between arterial oxygen pressure levels and inspired oxygen fraction (PaO2/FiO2), which classifies the severity of hypoxemia into mild (200 mm Hg to 300 mm Hg), moderate (100 mm Hg to 200 mm Hg) and severe (100 mm Hg) [6]. A modification to this predictor is the ratio of peripheral arterial oxygen saturation to inspired oxygen fraction (SaO2/FiO2), which is a noninvasive tool [7]. Diaphragmatic ultrasound is a widely available noninvasive, nonionizing imaging technique to directly assess diaphragm function using muscle thickening in the apposition and excursion zone of the diaphragm [8]. These measures could be predictors of mechanical ventilation in patients with acute respiratory failure who are not on rescue therapy or are close to respiratory arrest.

This study aims to propose and evaluate the use of a new non-invasive predictive index, based on the relationship between oxygenation and diaphragmatic function, for the requirement of invasive mechanical ventilation in patients with SARS-CoV-2 infection who are admitted to the emergency department.

## Method

### Study design

A prospective predictive cohort study was performed. Data were obtained from the emergency department of the Hospital Universitario Mayor-Méderi. This is an institution of high hospital complexity where 238,000 consultations are attended annually in the adult emergency room, attended in 110 observation beds.

All patients admitted to the emergency room of the Hospital Universitario Mayor with respiratory failure (not severe or in imminent respiratory arrest) and a confirmed diagnosis of SARS-CoV-2 pneumonia was included. The diagnosis of pneumonia was made by radiological imaging, and the diagnosis of SARS-CoV-2 infection was made by rt-PCR.

### Inclusion and exclusion criteria

#### Inclusion criteria

**Fig. 1:**
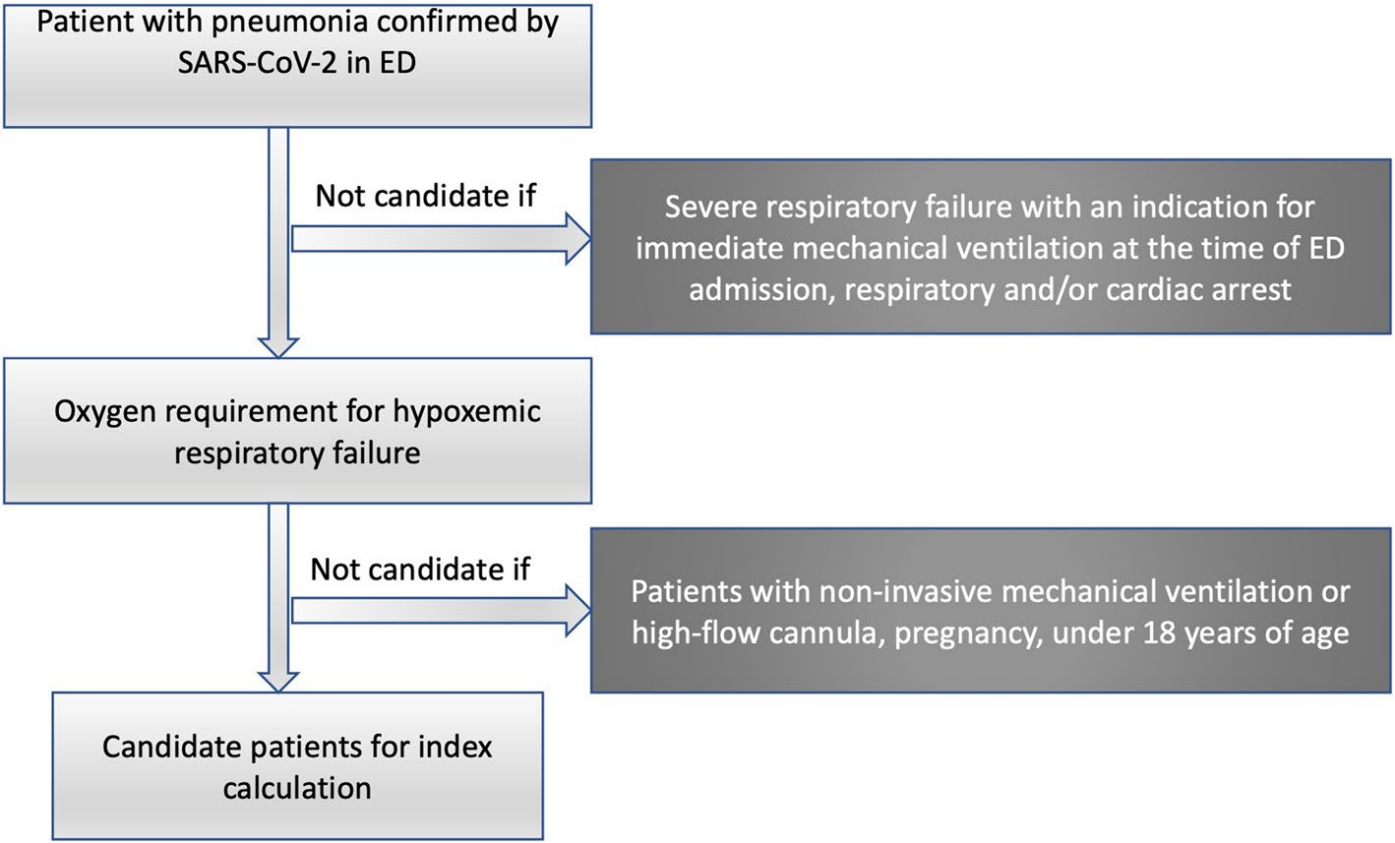
Flowchart for patient inclusion. ED: emergency department

Patients older than 18 years with a diagnosis of pneumonia by radiological imaging plus positive PCR test for SARS-CoV-2, who did not have advance directives such as an indication of no resuscitation, no orotracheal intubation, or hospitalization in the intensive care unit. All patients were admitted through the emergency department, no patient at the time of measurements had non-invasive mechanical ventilation or high-flow cannula, only patients with standard oxygen therapy were included, (Fig. 1).

#### Exclusion criteria

patients with severe respiratory failure with an indication for immediate mechanical ventilation at the time of ED admission, respiratory and/or cardiac arrest, pregnancy, unavailability of an emergency physician at the time of patient admission to the ED, history of lung tissue resection, history of lung transplantation.

### Sampling method

According to inclusion and exclusion criteria, this pilot study included 100 patients sequentially enrolled in the emergency department.

### Measurements

Patients presenting to the ED with a diagnosis of mild to moderate acute respiratory failure with SARS-CoV-2 pneumonia had measurements of diaphragmatic excursion and diaphragmatic thickening performed within the first 24 h after admission to the ED. Subsequently, the relationship between diaphragmatic excursion and SAFI was calculated, establishing an index called Ultrasound Diaphragmatic Excursion SO_2_/FiO_2_index (U.D.E.S.I) which was calculated as follows:

$$RI=\left(\frac{REXI}{SAFI}\right)100$$

$$LI=\left(\frac{LEXI}{SAFI}\right)100$$

RI: Right Index, REXI: Right Diaphragmatic Excursion, SAFI: ratio of saturation to inspired oxygen fraction (SO_2_/FiO_2_), LI: Left Index, LEXI: Left Diaphragmatic Excursion.

According to previously published recommendations, the manner of measuring right and left diaphragmatic excursion was performed [9]. B-mode was first used to find the best focus and to select the scan line of each hemidiaphragm. The liver was used as a window on the right while the spleen was used for the left hemidiaphragm. The same ultrasound equipment (SonoSite M-Turbo P08792/P09823) was used for all measurements, using a convex probe between the midclavicular and anterior axillary lines, in the subcostal area, and directed medially, cranially, and dorsally, so that the ultrasound beam reaches the posterior third of the hemidiaphragm perpendicularly. Subsequently, the M mode was used to quantify the difference in inspiration and expiration of each respiratory cycle. All patients were assessed in the semi-recumbent position.

All measurements were made in total by two emergency medicine specialists with basic training in the use of ultrasound. To obtain the ultrasound images, the emergency physician does not need extensive experience, only a certified basic training in ultrasound is enough to obtain the necessary measurements for the calculation of the index. The two specialists came to the patient's assessment at the same time, each one made two measurements of the left and right diaphragmatic excursion, later the values were averaged. Measurement values and that value was averaged with the data from the second observer and that final average was entered into the database. In the event that someone could not perform one of the measurements, only the average measurements found by one of the evaluators were recorded. Although the evaluation of the excursion of the left hemidiaphragm was the most difficult to obtain, these values were achieved in 100% of the patients.

### Statistical analysis

All analyses were performed using the statistical program R Version 4.2.0 © 2009–2021 RStudio, PBC. Before recording the information in the data collection base, a verification of the demographic variables was carried out again and the values registered in the database were confirmed with those obtained in the measurements in the field, this in order not to commit errors in data entry. For continuous variables, the Shapiro-wilks normality test was initially calculated and then, according to their distribution, expressed in terms of mean and standard deviation or median and interquartile range. Categorical variables were reported as absolute frequencies and percentages.

Subsequently, the diagnostic performance of the right and left indices were determined by creating areas under the curve (AUC). The ideal cutoff point for each index was also determined, and sensitivity and specificity calculations were performed for the mechanical ventilation outcome.

## Results

**Fig. 2:**
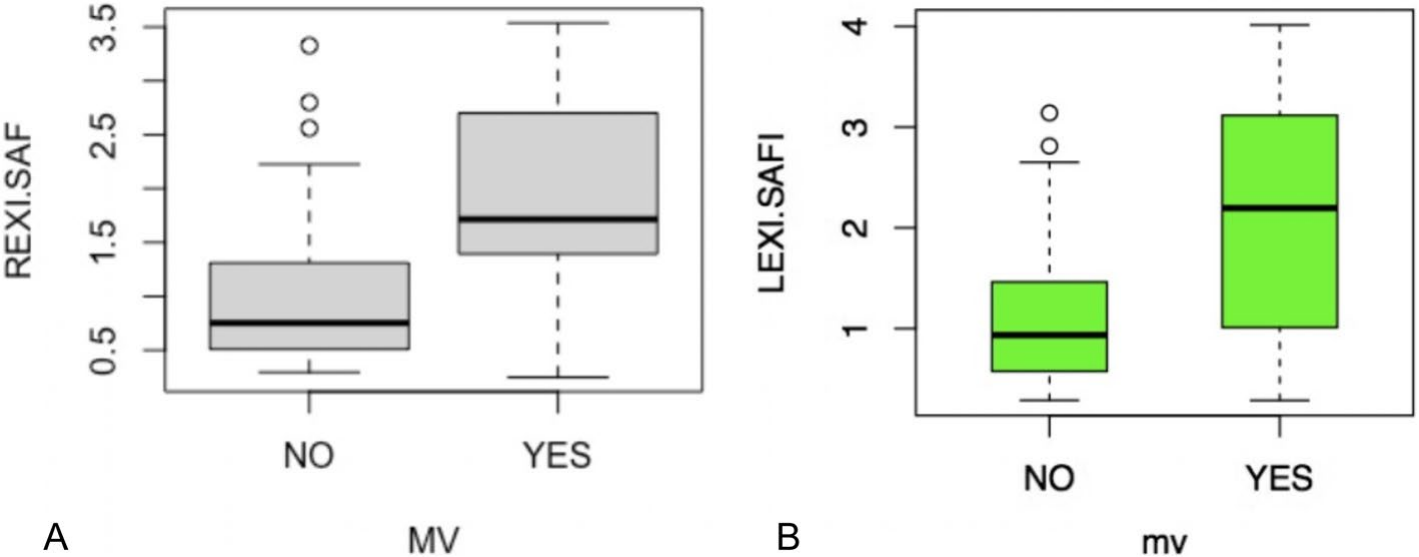
Right (**A**) and left (**B**) U.D.E.S.I according to mechanical ventilation outcome. MV: mechanical ventilation, REXI_SAF (ultrasound diaphragmatic excursion So2/FiO2 index right), LEXI_SAFI (ultrasound diaphragmatic excursion So2/FiO2 index left), A: *p* = 0.009, B: *p* = 0.026

Table 1 shows the characteristics of the population. It was documented that there was a difference in the age of the patients who required mechanical ventilation, with older patients presenting more frequently with this outcome. No differences were found in the sex of the patients or the history of mechanical ventilation outcome; the most frequent comorbidity was arterial hypertension, and the least frequent was asthma. Regarding the body mass index (BMI) of the population, an average of 28.02 kg/m^2^ was found, but with no significant difference between the groups. It was possible to document those patients with lower SAFI, PAFI, and ROX values significantly required mechanical ventilation. The ultrasound pattern of the pulmonary parenchyma showed no difference between the two groups. Additionally, it was documented that there was a significant difference in the values of both right and left diaphragmatic excursion between patients who required or did not require mechanical ventilation (Fig. 2). In addition, it was found that there was a positive correlation between the measured value of the excursion of the right and left diaphragm, however, this was not very high (0.501). We performed measurements of the fractional thickening of the diaphragm, however, we decided not to include them in the analyzes as more than 10% of the data were missing, additionally this measurement in the left diaphragm was very limited.

**Table 1:** Patient characteristics

*n* = 100			
**VARIABLE**	**MV(-)**	**MV( +)**	**P**
	74(74%)	*n* = 26(26%)	
AGE	61.45(31–88)	71.5(59–90)	< 0.001
SEX(F(%)/M(%))	35(79.5)/38(69.0)	9(20.4)/17(30.9)	0.4182
Chronic hypertension (%)	30(69.76)	13(30.23)	0.5433
DM(%)	19(65.51)	10(34.48)	0.3247
HF(%)	2(66.6)	1(33.3)	1.000
DISL(%)	2(33.3)	4(66.6)	0.06255
CRI(%)	6(75.0)	2(25.0)	1.000
HIPO(%)	11(73.33)	4(26.66)	1.000
COR_D(%)	5(83.33)	1(16.66)	0.9541
COPD(%)	5(45.45)	6(54.54)	0.05441
ASTHMA(%)	2(100)	0(0)	0.974
BMI	27.54(17.7–46.0)	28.52(22.2–41.6)	0.355
CREAT	1.16(0.07–12.54)	1.13(0.40–4.40)	0.915
UN	21.52( 6.30–80.20)	28.24(10.10–63.90)	0.03854
LACT	1.69( 1.00–3.80)	1.93(1.10–3.50)	0.09361
PAFI	185.16(57.00–397.00)	120.92(58.00–391.00)	< 0.001
SAFI	258.0(85.0–438.0)	162.5(84.0–438.0)	< 0.001
ROX	11.616(2.750–23.100)	6.833(2.340–24.340)	0.001249
LUNG_P(AB/B)	8(100)/66(71.73)	0(0)/26(28.26)	0.1843
MV_D	0	14.85(5.00–30.00)	NA
ICU_D	0	16.55( 5.00–32.00)	NA
IHX_D	8.027(2.00–20.00)	19.92(6.00–40.00)	< 0.001
MORT(%)	0	19(73.0)	< 0.001
REXI	1.66(1.44–2.192)	2.23(1.86–2.732)	0.009
LEXI	1.85(1.55–2.57)	2.49(2.06–3.34)	0.02
UDESI-R	0.9637(0.2962–3.3277)	1.8286(0.2477–3.5379)	< 0.001
UDESI-L	1.0929( 0.2876–3.1412)	2.1041( 0.2865–4.0146)	< 0.001

*SEX F* Feminine /, *M* Male, *MV* Mechanical ventilation, *DM* Diabetes mellitus, *HF* Heart failure, *DISL* Dyslipidemia, *CRI* Chronic renal failure, *HIPO* Hypothyroidism, *COR_D* Coronary heart disease, *COPD* Chronic obstructive pulmonary disease, *BMI* ﻿Body-mass index, *CREAT* Creatinine, *UN* Ureic nitrogen, *LACT* Lactate, *PAFI* ﻿Pao2/Fio2 mm Hg, *SAFI* SaO2/FiO2, *ROX*: Respiratory rate oxygenation, *LUNG_P* Lung pattern, *MV_D* Days of mechanical ventilation, *ICU_D* Days of intensive care unit, *IHX_D* Days of hospitalization, *MORT* Mortality, *REXI* Right ultrasound diaphragmatic excursion, *LEXI* Left ultrasound diaphragmatic excursion, *UDESI-R* Ultrasound diaphragmatic excursion So2/FiO2 index right, *UDESI-L* Ultrasound diaphragmatic excursion So2/FiO2 index left

In-hospital mortality in the study population was 19%, and all patients who died required mechanical ventilation. Regarding in-hospital mortality, a significant association was found with age, LEXI/SAFI and REXI/SAFI with an OR of 1.10 (CI95%:1.03–1.20), 2.46(CI:95%1.35–4.88) and 2.94(CI:95%1.46–6.49) respectively.

Analyses were performed to establish the diagnostic performance of the test for the prediction of mechanical ventilation. It was found that, in general, the test for the right index showed a specificity of 82.4% with a sensitivity of 76.9%, a negative predictive value of 91.0, and a positive predictive value of 60.6. Likewise for the left index, an overall specificity of 90.5% was found with a sensitivity of 65.3%, a negative predictive value of 88.1 and a positive predictive value of 70.8. The ideal cut-off point for the right index was 1.485, and For the left index, the threshold point was 1.856 (Table 2). Finally, analyzes were performed to establish the diagnostic performance of all the tests for the prediction of mechanical ventilation (Table 2). It was documented that the highest sensitivity had the ROX (0.80), but without much difference compared to SAFI, PAFI and the right U.D.E.S.I. While the highest specificity was documented in the left and right U.D.E.S.I indices (0.90, 0.82, respectively).

**Table 2:** Diagnostic performance of diaphragm ultrasound for mechanical ventilation

Variable	Thresholds	Se	SP	NPV	PPV	AUC
REXI	2.015	0.659	0.689	0.85	0.425	0.683(0.564–0.801)
LEXI	2.3	0.653	0.648	0.842	0.395	0.672(0.552–0.791)
ROX	6.09	0.807	0.689	0.910	0.477	0.727)(0.604–0.850)
SAFI	156.6	0.769	0.716	0.898	0.487	0.733(0.602–0.863)
PAFI	87	0.769	0.783	0.906	0.555	0.741(0.608–0.874)
U.D.E.S.I.r	1.485	0.769	0.824	0.910	0.606	0.798(0.676–0.920)
U.D.E.S.I.l	1.856	0.653	0.905	0.881	0.708	0.793(0.674–0.911)

*Se* Sensitivity, *SP* Specificity, *PPV* Positive predictive value, *NPV* Negative predictive value, *AUC* Area under receiver operating characteristic curve, *PAFI* ﻿Pao2/Fio2 mm Hg, *SAFI* SaO2/FiO2, *ROX* Respiratory rate-oxygenation, *REXI* Right ultrasound diaphragmatic excursion, *LEXI* Left ultrasound diaphragmatic excursion, *U.D.E.S.I.r* Ultrasound diaphragmatic excursion So2/FiO2 index right, *U.D.E.S.I.l* Ultrasound diaphragmatic excursion So2/FiO2 index left

**Fig. 3:**
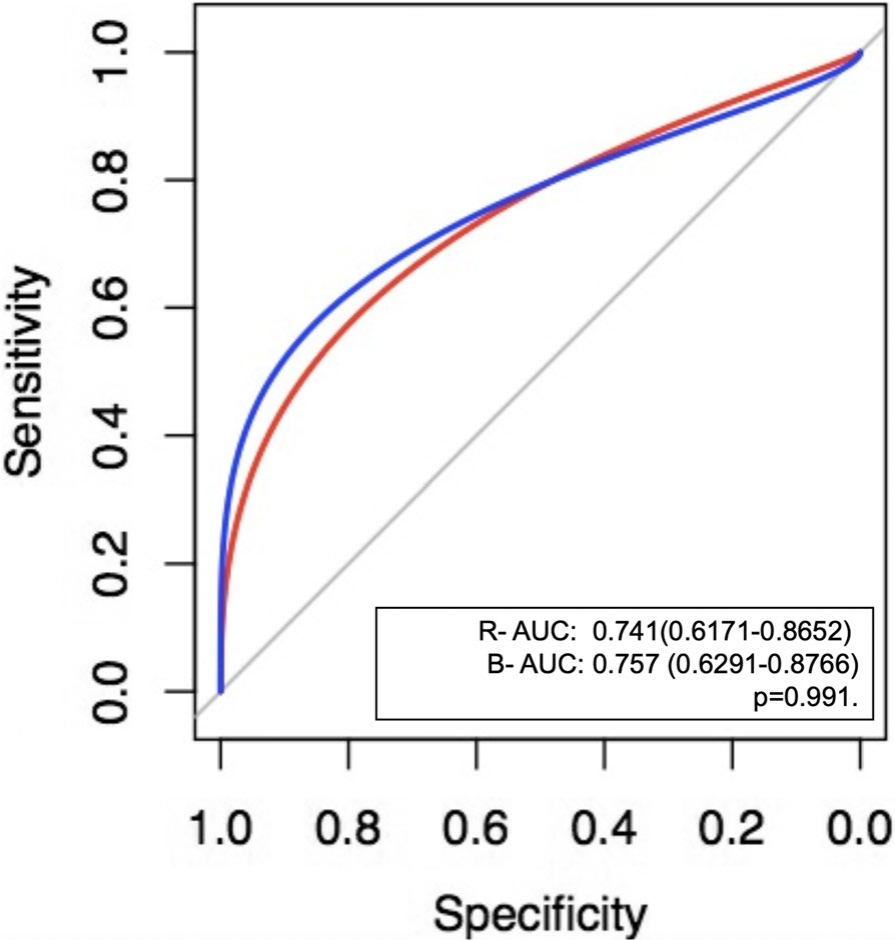
Receiver operating characteristic curves comparing prediction tools. REXI_SAF (ultrasound diaphragmatic excursion So2/FiO2 index right), LEXI_SAFI (ultrasound diaphragmatic excursion So2/FiO2 index left), SAFI: SaO2/FiO2, PAFI: ﻿Pao2/Fio2 mm Hg, ROX: respiratory rate-oxygenation

Figure 3 shows the area under receiver operating characteristic curve of the evaluated predictors, the AUC values of the ROC curve of the right and left U.D.E.S.I were the highest (0.798–0.793), however, a significant difference was only demonstrated between ROC curve AUC of right U.D.E.S.I compared to PAFI (*p* = 0.048), no other significant differences between ROC AUC were documented.

## Discussion

This work aims to evaluate another way of establishing a relationship between oxygenation and the patient's respiratory work in a non-invasive manner; therefore, the So2/FiO2 ratio was chosen as an indicator of oxygenation and was related to the diaphragmatic excursion measured by ultrasound as a way of evaluating the patient's respiratory effort. We calculated the diaphragm excursion on both the right and left sides and calculated the index as previously reported in this paper.

In this cohort it was shown that low SAFI and PaO2/FiO2 values were associated with the need for mechanical ventilation. The average SAFI of the cohort was 233 (minimum 84 and maximum 438), the SAFI value of 156 was the cutoff point for patients to need mechanical ventilation, a value that is very similar to previous reports where saturation/fraction of inspired oxygen (SAFI) of 158 ± 32 was associated with mortality from SARS COVID-19 (OR: 0.94, 95% CI: 0.91–0.97) [10]. As for PaO2/FiO2, it was documented that values lower than 87 were related to the need for mechanical ventilation. This data is important since the average PaO2/FiO2 of the population was 168, a value that is well below the international recommendations on the indications for mechanical ventilation and ARDS [6] but more in accordance with expert recommendations to take into account PAFI lower than 150 when making the decision to intubate in this group of patients with SARS COV2 infection [11]. This is probably because the cohort was collected in an institution located in a city at an altitude of 2600 m above sea level.

The relationship between So2 and respiratory frequency (ROX index) was another variable associated with the outcome of mechanical ventilation. The average value was 10.32, with a cut-off point of 6.09 for the need for mechanical ventilation. This data no differs significantly from previous reports where the ROX index of < 25.26 on day 1 of hospital stay was associated with the outcome of mechanical ventilation, the sensitivity of 90.2%, and the specificity of 75% [12]. Further, as in other reports, it can be used as a predictor of intubation and mechanical ventilation [12–14], especially as an indicator of early failure of mechanical ventilation [15].

Interestingly, our work showed that the value of both right and left diaphragmatic excursion were significantly different between the groups that required and did not require mechanical ventilation, a result similar to that found in a recently published study [16], however, the value of the area under the curve of our work was not as significant as that shown in the study by Helmy et al. [16], who showed a value of 0.96 (0.85–1.00) and 0.94 (0.82–0.99) for the right and left diaphragm, respectively; in our study, the AUC was 0.683 (0.56–0.80) and 0.67 (0.55–0.79), much lower, it is likely that this is due to the fact that the study population by Helmy et al. [16] included patients admitted to the ICU with severe COVID, while our study included patients admitted to the emergency room regardless of the severity of the COVID infection.

When reviewing the prognostic performance of ROX in terms of predicting the requirement for mechanical ventilation, the results are mixed; there are reports of sensitivity with high figures of 90–96% with a specificity of 62–75% [12–17], and when evaluating the AUC of ROX to predict mechanical ventilation, values of 0.727 (0.634–0.821) [13]. In our population, the ROX had a sensitivity of 0.80 and a specificity of 0.68 with an AUC of 0.727, similar to previous reports described in the literature, but when comparing the ROX with the U.D.E.S.I it was possible to determine that the sensitivity, although slightly lower ( 0.76 for the right and 0.65 for the left) was more or less similar, however, the specificity was much higher (0.82 for the right and 0.90 for the left), likewise the AUC of UDESI was higher (0.798 for the right and 0.793 for the left). Which shows the advantage of U.D.E.S.I over ROX for the prediction of mechanical ventilation requirement.

We decided to use SAFI instead of PAFI as a tool for assessing oxygenation because it is a non-invasive measure that is easy to perform in all emergency services. SAFI proved itself to be capable of predicting the need for mechanical ventilation in our population. however, by adding diaphragmatic activity as a tool to assess respiratory effort through the U.D.E.S.I index, it was possible to document that adding the ultrasound variable to the oxygenation variable improves the specificity in the prediction while preserving the sensitivity values. In the sample studied, an important difference was documented in the positive predictive value of the U.D.E.S.I and SAFI (0.708 and 0.487), with similar negative predictive values (0.881 and 0.898).

In our population, overall mortality was 19%, all of them were part of the group of ventilated patients, which allowed us to conclude that mortality among ventilated patients was 73%, data similar to those previously reported where up to 25% of the need for mechanical ventilation was documented, with mortality rates in hospitalized patients ranging from 3,6% to 34,6% [3].

The U.D.E.S.I. is a tool with non-invasive measurements that aims to assess the patient's oxygenation status and work of breathing by evaluating the excursion of the diaphragm by ultrasound. It may be an alternative for the classification of patients with respiratory failure secondary to SARS COVID-19 infection, as it could help differentiate more objectively within the group of patients with hypoxemia, those who are in respiratory distress and will probably need a ventilator mechanic as support therapy in the medical management of viral pneumonia by COVID 19.

The results of this work justify the design of a study with a larger sample to prospectively validate the score with a significant sample.

## Limitation

This study has several limitations, it is a pilot study, and therefore no sample size calculation was performed. It was completed in a single institution, which could affect the homogeneity of the population. However, patients were collected sequentially as they behave in real life.

The results of this work do not allow changing clinical practice, given the evident limitations of a pilot study with a small sample from a single institution. We did not have patients with pleural effusion, so we do not know the behavior of the index in patients with this alteration. However, the results of this work allow justifying the design of a more extensive validation study of the score that allows obtaining better conclusions from the statistical point of view.

## Conclusion

The relationship of So2/FiO2 and diaphragm excursion measured by both right and left ultrasound could predict the need for mechanical ventilation of the patient with COVID-19 pneumonia in the emergency room and could constitute a valuable tool since it uses noninvasive parameters and is easily applicable at the patient’s bedside. However, a more extensive study is needed to validate these preliminary results.

## Data Availability

The datasets used and/or analyzed during the current study are available from the corresponding author on reasonable request.

## Abbreviations

SAFI: Ratio of peripheral arterial oxygen saturation to inspired oxygen fraction
U.D.E.S.I: The ultrasound diaphragmatic excursion So2/FiO2 index
ARDS: Acute Respiratory Distress Syndrome
PaO2/FiO2: Relationship between arterial oxygen pressure levels and inspired oxygen fraction
RI: Right Index
REXI: Right Diaphragmatic Excursion
LI: Left Index
LEXI: Left Diaphragmatic Excursion.
ROX index: The relationship between pulse oximetry on the fraction of inspired oxygen and respiratory rate
